# Multimodal Sensing and Digital Biomarkers in Virtual Reality-Based Cognitive Rehabilitation: Clinical Validation, Adaptive Systems, and Real-World Translation

**DOI:** 10.3390/s26206479

**Published:** 2026-10-13

**Authors:** Francesco Rundo, Sabrina Musso, Serafino Buono, Marilena Recupero, Raffaele Ferri

**Affiliations:** 1 Oasi Research Institute—IRCCS, 94018 Troina, Italy; frundo@oasi.en.it (F.R.); smusso@oasi.en.it (S.M.); fbuono@oasi.en.it (S.B.); mrecupero@oasi.en.it (M.R.); 2 Department of Medicine and Surgery, University of Enna “Kore”, 94100 Enna, Italy

**Keywords:** virtual reality, cognitive rehabilitation, multimodal sensing, digital biomarkers, adaptive rehabilitation, indoor assisted living

## Abstract

**Highlights:**

**What are the main findings?**

- Multimodal sensing allows virtual reality rehabilitation to capture not only task performance but also gaze, movement, autonomic responses, and neural activity, providing information on patients’ strategies, effort, engagement, and learning.
- Behavioral and interaction-derived measures are currently the most established, whereas most sensor-derived variables remain candidate biomarkers and physiologically driven closed-loop systems are still experimental.

**What are the implications of the main findings?**

- Multimodal measures should be clinically interpretable and linked to explicit rehabilitation decisions, such as adjusting task difficulty, pacing, guidance, or feedback.
- Translation into indoor assisted living requires multicenter validation, evidence of transfer to everyday functioning, and safe integration with remote supervision, wearable devices, and ambient home sensing.

**Abstract:**

Virtual reality (VR) is evolving from a platform for cognitive exercises into an instrumented rehabilitation environment capable of simultaneously delivering treatment and quantifying behavior. This critical narrative review examines how behavioral, ocular, kinematic, physiological, and neurophysiological signals can be transformed into clinically meaningful digital measures, candidate biomarkers, adaptive interventions, and real-world functional outcomes. PubMed/MEDLINE, Scopus, and IEEE Xplore were searched from database inception to 20 September 2026, supplemented by backward and forward citation searching and targeted searches. Clinical, methodological, validation, and directly relevant technical studies were considered, including studies in healthy participants when methodologically informative. Of 690 records identified, 145 duplicates were removed; 545 unique records were screened and 303 formed the candidate evidence pool for critical narrative appraisal. Behavioral and interaction-derived measures are the most established, while eye tracking, kinematic sensing, autonomic monitoring, EEG, and fNIRS provide complementary information on attention, strategy, workload, and engagement. Quantitatively, combined hand- and eye-tracking features distinguished mild cognitive impairment from healthy controls with 93.3% accuracy, 100% sensitivity, and 83.3% specificity, while immersive VR life-skills training produced real-world functional gains of d = 1.00 post-training and d = 1.12 at 1-week follow-up. However, most sensor-derived variables remain digital measures or candidate biomarkers rather than clinically validated biomarkers; physiology-informed closed-loop adaptation is uncommon, and evidence that such adaptation produces sustained ADL or assisted-living benefits is lacking. Progress now depends on standardized reporting, external and multicenter validation, clinically interpretable adaptive models, and objective demonstration of real-world transfer.

## 1. Introduction

Virtual reality (VR) has progressively evolved from a technology used mainly to deliver simulated exercises into an interactive and instrumented environment in which cognitive performance can be trained, observed, and quantified under standardized yet ecologically meaningful conditions. Earlier applications emphasized immersion, engagement, and repetition; more recent systems combine realistic activities of daily living (ADL) with embedded tracking, automated task logging, and adaptive algorithms. This evolution is particularly relevant to cognitive rehabilitation, where the objective is not only to improve performance in isolated neuropsychological tasks but also to restore functional competence in complex everyday situations. Reviews of VR-based neuropsychological assessment and rehabilitation have highlighted its potential to bridge the gap between controlled testing and real-world behavior, while also identifying substantial heterogeneity in devices, tasks, and outcomes [1,2,3,4].

Conventional cognitive and functional outcome measures remain essential, but they have important limitations. Paper-and-pencil tests and global rating scales generally provide brief, episodic observations obtained in highly structured settings; they may therefore be relatively insensitive to fluctuations, compensatory strategies, learning trajectories, and subtle changes in the organization of behavior. Moreover, performance in traditional tests does not always predict how independently a person can plan, sequence, and complete instrumental activities of daily living (IADL). Ecologically oriented assessments improve this correspondence, but they are often time-consuming, difficult to standardize, and dependent on examiner judgment [5,6]. VR can complement, not replace, these measures by recording how a task is performed, rather than only whether it is completed successfully.

A central challenge in cognitive rehabilitation is that similar levels of observable task performance may arise from different underlying mechanisms. Successful task completion may reflect efficient cognitive processing, but it may also depend on compensatory strategies, increased effort, repeated self-correction, or external support. Conversely, impaired performance may result from cognitive dysfunction, motor or sensory limitations, fatigue, anxiety, or unfamiliarity with the interface. Consequently, a single behavioral outcome may be insufficient to characterize the processes underlying rehabilitation performance or to determine how an intervention should be adapted. Multimodal sensing addresses this limitation by combining complementary information about what the patient achieves, where attention is directed, how actions are organized, how much physiological effort is required, and which neural systems are engaged. Its theoretical value therefore lies not in sensor accumulation per se, but in resolving otherwise ambiguous performance states and linking them to clinically interpretable rehabilitation decisions.

During a virtual rehabilitation session, continuous information can be derived from response accuracy, error type, reaction time, action sequences, navigation paths, requests for assistance, and changes across repeated trials. Head, hand, and whole-body tracking may characterize movement efficiency, hesitation, coordination, and cognitive–motor integration, whereas eye tracking and pupillometry can provide indices of visual exploration, attention allocation, and workload. Wearable physiological sensors may capture heart rate variability, electrodermal activity, and respiration, while electroencephalography (EEG), functional near-infrared spectroscopy (fNIRS), and neuroimaging can contribute measures of neural engagement, plasticity, and treatment responsiveness. Studies combining VR with kinematic or oculomotor analysis, machine learning, EEG, and neuroimaging illustrate the possibility of deriving multidimensional digital markers from rehabilitation itself [7,8,9,10,11,12,13]. However, a digital measure should not be considered a clinically meaningful biomarker merely because it is automatically recorded; reliability, sensitivity to change, external validation, interpretability, and association with relevant clinical or functional outcomes remain necessary.

These developments also create opportunities for home-based rehabilitation and indoor assisted living. Our previous studies showed that virtual training can target functional living skills remotely in adolescents and young adults with intellectual disability and can support the relearning and, in some cases, generalization of everyday skills in people with major neurocognitive disorders [14,15,16]. More recent reviews from our group have emphasized both the promise of VR across acquired cognitive disorders and the unresolved implementation challenges of immersive technologies in mild cognitive impairment and dementia, including usability, standardization, scalability, and demonstration of functional transfer [3,17]. In assisted-living settings, sensor-integrated VR could support longitudinal monitoring, remote supervision, and individualized adjustment of task difficulty, but only if technological complexity is balanced against accessibility, safety, privacy, and clinical usefulness.

Accordingly, the aim of this critical narrative review is to examine how multimodal sensing can be translated into clinically meaningful information within VR-based cognitive rehabilitation. Specifically, we consider the progression from acquisition of behavioral, ocular, motor, physiological, and neurophysiological signals, through feature extraction and development and validation of candidate digital biomarkers, to their potential use for individualized and adaptive rehabilitation. We further examine whether these approaches have demonstrated transfer beyond the virtual task to activities of daily living and other real-world functional outcomes, including their potential application in indoor assisted-living settings.

## 2. Literature Search and Scope of the Review

This work was designed as a critical narrative review supported by a structured and reproducible literature-identification strategy. It was not intended as a systematic review or meta-analysis, because its objective was to integrate heterogeneous clinical, methodological, technological, and conceptual evidence spanning different stages of multimodal VR-based cognitive rehabilitation.

PubMed/MEDLINE, Scopus, and IEEE Xplore were searched from database inception to 20 September 2026. The search combined three conceptual domains: (1) virtual, augmented, mixed, or extended reality; (2) cognitive rehabilitation, cognitive training, cognitive remediation, functional cognition, or neuropsychological rehabilitation; and (3) sensing and data-analysis technologies, including behavioral and interaction measures, eye tracking, pupillometry, motion and kinematic tracking, wearable sensors, physiological and neurophysiological signals, digital biomarkers, machine learning, artificial intelligence, multimodal integration, and adaptive or closed-loop systems.

In PubMed/MEDLINE, the search combined terms related to virtual reality (“Virtual Reality”[Mesh], “virtual reality”[tiab], “extended reality”[tiab], “mixed reality”[tiab], “augmented reality”[tiab], “immersive technology”[tiab], “virtual environment”[tiab], and “head-mounted display”[tiab]) with cognitive-rehabilitation terms (“Cognitive Remediation”[Mesh], “Cognitive Training”[Mesh], “cognitive rehabilitation”[tiab], “cognitive remediation”[tiab], “cognitive training”[tiab], “cognitive retraining”[tiab], “neuropsychological rehabilitation”[tiab], “functional cognition”[tiab], “functional living skills”[tiab], and “cognitive intervention”[tiab]) and sensing/analysis terms including sensor, biosensor, wearable, “digital biomarker”, “digital phenotype”, “behavioral data”, “interaction data”, “eye tracking”, gaze, pupillometry, kinematic, “motion tracking”, “motion capture”, “inertial measurement unit”, physiological, psychophysiological, ECG, PPG, “heart rate variability”, electrodermal, “skin conductance”, EEG, fNIRS, multimodal, “sensor fusion”, “machine learning”, “artificial intelligence”, adaptive, and “closed loop”.

In Scopus, the corresponding terms were searched in titles, abstracts, and keywords using TITLE-ABS-KEY, combining the same three domains with Boolean AND operators. In IEEE Xplore, the equivalent concepts were searched in All Metadata. Because IEEE Xplore limits the number of wildcard operators, singular/plural and related lexical variants were entered explicitly rather than through unrestricted truncation. The IEEE search included, among others, “virtual reality”, “extended reality”, “mixed reality”, “augmented reality”, “cognitive rehabilitation”, “cognitive training”, “functional cognition”, sensor, sensors, wearable, wearables, “digital biomarker”, “eye tracking”, gaze, pupillometry, kinematic, “motion tracking”, EEG, fNIRS, ECG, PPG, HRV, multimodal, “sensor fusion”, “machine learning”, “artificial intelligence”, adaptive, adaptation, “closed loop”, and “closed-loop”.

Peer-reviewed journal articles and full conference papers were considered. Eligible publications included clinical studies of VR-based cognitive rehabilitation or training; methodological and validation studies of sensor-integrated VR systems; studies of sensor-derived measures potentially relevant to rehabilitation monitoring, personalization, or functional transfer; and technical, protocol, review, or conceptual publications when they provided directly relevant information on signal acquisition, multimodal integration, biomarker development, adaptive intervention design, or implementation. Studies involving healthy participants were retained when they addressed methodological questions directly applicable to cognitive rehabilitation. Predominantly motor rehabilitation studies were considered only when they provided transferable information relevant to multimodal sensing, cognitive–motor integration, adaptive intervention, embodiment, or home-based monitoring. Conference abstracts or posters, books or book chapters, retractions and corrections, and publications without sufficient relevance to the predefined scope were excluded.

The three database searches identified 690 records (PubMed/MEDLINE, n = 251; Scopus, n = 283; IEEE Xplore, n = 156). Records were deduplicated using DOI, PMID, IEEE document identifier, and normalized bibliographic title, resulting in the removal of 145 duplicates. The remaining 545 unique records underwent title and abstract relevance screening according to the predefined scope of the review. Of these, 242 were excluded and 303 were retained as the candidate evidence pool for critical narrative appraisal. Initial relevance screening was performed by one author and independently verified by a second author; disagreements were resolved by discussion and consensus. The literature-identification process is summarized in Figure 1.

Database searching was supplemented by backward reference screening of relevant reviews and primary studies, forward citation searching of key publications, and targeted searches addressing specific technologies, clinical populations, validation frameworks, and emerging applications. These supplementary searches were used to support conceptual completeness and were not incorporated into the numerical database-flow denominator.

The 303 records retained after relevance screening constituted the candidate evidence pool for the narrative synthesis. Because these publications covered heterogeneous purposes and designs, Table 1 presents a structured comparison of representative studies selected to illustrate the major stages of the translational pathway, rather than an exhaustive listing of all retained records.

Taken together, the studies summarized in Table 1 show substantial attrition across this translational pathway. Technical feasibility and within-sample discrimination are relatively well represented, whereas independent clinical validation of sensor-derived biomarkers remains uncommon. Several behavioral, ocular, kinematic, EEG, and multimodal measures have shown associations with cognitive status or treatment-related change, but most remain digital measures or candidate biomarkers rather than clinically validated biomarkers. Fewer studies have used measured patient states prospectively to modify rehabilitation, and physiology- or neurophysiology-informed closed-loop adaptation remains particularly limited. Finally, although some studies demonstrate home deployment or transfer to real-world functional tasks, evidence that biomarker-informed closed-loop adaptation itself produces sustained improvements in ADL or assisted-living outcomes is currently lacking.

The organization of the review follows a translational sequence from signal acquisition to clinical application. Multimodal behavioral, ocular, motor, physiological, and neurophysiological signals are first transformed into extracted features and digital measures; these measures may subsequently qualify as candidate digital biomarkers if they show a reproducible relationship with clinically meaningful processes. Only after adequate analytical and clinical validation can such information plausibly support patient-state estimation and prospective adaptation of rehabilitation. The final translational step is demonstration that adaptive intervention produces benefits extending beyond the trained virtual task to everyday functioning, ADL, and potentially home or assisted-living environments. Importantly, evidence supporting one stage of this sequence should not be interpreted as evidence that the entire pathway has been validated.

## 3. Multimodal Sensing in VR Cognitive Rehabilitation

VR rehabilitation generates clinically relevant information across three complementary domains: behavioral and interaction-derived performance, ocular and kinematic behavior, and physiological or neurophysiological responses. The value of these signals depends on whether they capture interpretable aspects of cognition, effort, or adaptation.

### 3.1. Behavioral and Interaction-Derived Data

The most accessible data are generated directly by the virtual task. Accuracy, omissions, commission errors, response time, task-completion time, and the number of successfully completed steps provide conventional performance indices in a more granular format. Their interpretation becomes richer when the system also records error type, the stage at which the error occurred, whether it was spontaneously corrected, and the amount of prompting required. Two patients may achieve the same final score, although one proceeds efficiently while the other depends on repeated cues, retraces previous steps, or completes the task through trial and error. Such differences may reveal impaired planning, working memory, monitoring, or cognitive flexibility.

In ecologically oriented tasks, navigation trajectories and action sequences are particularly informative. Path length, unnecessary detours, repeated visits to the same location, inactivity, or deviations from an efficient sequence may characterize spatial disorientation, indecision, perseveration, or ineffective problem solving. Virtual shopping and other IADL have therefore been used to derive multidimensional behavioral profiles and machine-learning classifications rather than simple pass-fail outcomes [7,26]. Similarly, our studies of virtual functional living skills training recorded task acquisition and the need for assistance across repeated sessions in people with intellectual disabilities or major neurocognitive disorders, illustrating how performance histories can document learning and maintenance in clinically relevant activities [14,15,16].

Repeated measurement also permits estimation of learning curves and intraindividual variability. The slope of improvement, the point at which performance reaches a plateau, and the stability of gains may be more informative than the final score alone. Trial-to-trial variability may indicate fluctuating attention, fatigue, inconsistent strategy use, or reduced cognitive control; decreasing variability may instead signal consolidation. Help requests and automated prompts can be analyzed as markers of the level and type of support required. However, behavioral measures remain task-dependent: longer completion time may reflect cognitive slowing, cautious accuracy, interface unfamiliarity, or motor impairment. Interpretation should therefore consider task structure, motor demands, previous technology experience, and the simultaneous sensor context.

### 3.2. Eye, Motion, and Kinematic Sensing

Eye tracking adds information about what the patient attended to before an action was taken. Fixation duration and distribution, saccadic transitions, revisits to inspected objects, gaze entropy, and time spent looking at relevant or distracting stimuli can characterize visual search, selective attention, uncertainty, and planning. Pupillary changes may provide an additional, although nonspecific, indicator of cognitive effort or arousal. In a virtual kiosk task, combined hand- and eye-movement features distinguished individuals with mild cognitive impairment from healthy participants, supporting their potential as digital markers of subtle functional inefficiency [8]. Eye tracking can also become part of the intervention: gaze-contingent systems can detect attention drifting toward distractors and trigger feedback intended to restore task focus, as demonstrated in VR attention training for adults with attention-deficit/hyperactivity disorder [41].

Motion sensing complements gaze by showing how intention is translated into action. Head and hand trajectories can quantify reach efficiency, movement onset, hesitation, corrective submovements, spatial accuracy, and coordination between visual exploration and object manipulation. Whole-body tracking may additionally capture posture, balance, orientation, and cognitive–motor integration. These data can be obtained from controllers, optical tracking, depth cameras, motion-capture systems, hand-tracking cameras, or inertial measurement units. Devices such as the Leap Motion Controller have been combined with cognitive VR tasks and EEG to relate interaction performance to attention-related brain activity [42].

Kinematic abnormalities should not automatically be interpreted as cognitive deficits. Slower or less direct movement may result from weakness, tremor, bradykinesia, pain, visual impairment, or limited familiarity with controllers. Conversely, adequate task accuracy may conceal inefficient scanning or repeated corrective movements. Joint analysis of gaze and motion can help distinguish failure to detect a target from difficulty planning or executing the response. Calibration quality, tracking loss, occlusion, controller latency, and hand dominance must also be reported because measurement error can resemble clinical variability.

### 3.3. Physiological and Neurophysiological Sensing

Physiological signals provide information about the internal cost of performance that cannot be inferred reliably from task accuracy alone. Electrocardiography (ECG) and photoplethysmography can provide heart rate and heart rate variability indices; electrodermal activity reflects sympathetic arousal; and respiratory rate and pattern may indicate effort, stress, or relaxation. When synchronized with task events, these signals may identify excessive challenge, disengagement, fatigue, or autonomic reactivity. This matters when similar performance conceals substantially different physiological effort. Physiological monitoring has consequently been proposed as a basis for identifying a “just-right” level of VR task difficulty and eventually adapting the intervention in real time [20,21,43].

These measures are nevertheless indirect and context-sensitive. Increased heart rate or electrodermal activity may represent cognitive workload, anxiety, novelty, physical movement, discomfort, or cybersickness. Heart-rate variability is influenced by respiration, posture, medication, age, and cardiovascular health. Physiological features should therefore be interpreted relative to an individual baseline, synchronized with behavioral events, and preferably combined with other modalities rather than treated as stand-alone indicators of cognitive state.

EEG and fNIRS extend sensing to cortical activity. EEG offers millisecond-level temporal resolution and can characterize spectral power, event-related responses, functional connectivity, and states associated with attention, memory, workload, or engagement. VR-EEG systems have been applied to scene-based memory rehabilitation in mild cognitive impairment [10], to monitor responses in severe disorders of consciousness [19], and to examine neuroplastic changes following VR cognitive training after stroke [11]. fNIRS provides a more portable measure of cortical hemodynamic activity than functional magnetic resonance imaging and may be more compatible with naturalistic VR interaction, although both fNIRS and EEG remain vulnerable to motion artifacts and require careful synchronization and preprocessing. Neuroimaging and electrophysiology may ultimately help identify predictors of treatment response rather than merely documenting post-treatment change [9,44].

Together, behavioral, ocular, kinematic, physiological, and neurophysiological signals describe complementary levels of rehabilitation: what the patient accomplishes, where attention is directed, how actions are organized, how much effort is required, and which neural systems are engaged. Their greatest value is likely to emerge from clinically interpretable combinations linked to specific task events and functional outcomes, rather than from indiscriminate sensor accumulation.

## 4. From Sensor Signals to Digital Biomarkers

The availability of continuous data does not, by itself, make VR-based rehabilitation clinically informative. Its added value depends on transforming heterogeneous sensor outputs into measures that represent specific cognitive processes, can be reproduced, and change in ways relevant to treatment. This is especially important in VR, where performance is influenced by cognition, motor ability, sensory function, familiarity with technology, motivation, and task design.

### 4.1. Candidate Digital Biomarkers

Behavioral and interaction-derived features can provide candidate markers of attention and executive control. Missed targets, distractor selections, inefficient action sequences, repeated corrections, prolonged pauses, and reliance on prompts may reflect impaired selective attention, monitoring, planning, or cognitive flexibility. Working memory may be reflected by failure to retain intermediate goals, repetition of completed steps, loss of task sequence, or declining accuracy as memory load increases. In virtual instrumental activities, path length, revisiting behavior, directional errors, and search time can characterize spatial navigation and prospective planning. Processing speed may be represented by reaction or completion time, but these measures require consideration of motor slowing, visual search, and interface demands.

Ecologically oriented VR studies illustrate the potential of combining several features rather than relying on a single score. Cavedoni et al. proposed integrating kinematic measures recorded during virtual instrumental activities with clinical and neuropsychological data to identify multidimensional patterns of mild cognitive impairment [7]. In a virtual kiosk task, Kim et al. found that hand-movement speed, fixation duration, completion time, and errors differed between participants with mild cognitive impairment and healthy controls, supporting the value of combined motor, oculomotor, and behavioral features [8]. Immersive virtual-supermarket studies have likewise combined navigation, task, and interaction variables with machine-learning models to identify early neurocognitive impairment or estimate cognitive status [25,26].

Cognitive–motor integration can be examined through the temporal relationship between gaze, decisions, and movement. Delayed movement initiation after target fixation may suggest planning difficulty, whereas repeated corrective movements despite adequate visual exploration may indicate inefficient motor execution. Physiological and neurophysiological data may add markers of workload, fatigue, arousal, and engagement. Heart-rate variability, electrodermal activity, respiration, pupillary responses, and EEG features may indicate whether successful performance required excessive effort or whether deterioration reflects fatigue. EEG-integrated VR studies have examined scene-memory rehabilitation in mild cognitive impairment, multimodal responses to rehabilitation tasks in older adults, and training-related neuroplastic changes after stroke [10,11,18,44]. Brain imaging may also identify baseline patterns associated with treatment responsiveness, shifting sensing from documenting change to predicting who is likely to benefit [9].

Repeated sessions generate longitudinal markers. The rate of improvement, reduction in prompts, stabilization of performance, tolerance of increasing complexity, and retention after treatment interruption may quantify learning and consolidation. A treatment-response biomarker should ideally detect meaningful change before it becomes evident on conventional scales and distinguish genuine learning from practice effects or increasing familiarity with the interface.

### 4.2. Clinical Validation

A clear hierarchy is required. A raw sensor signal is the unprocessed recording, such as an EEG voltage trace, gaze coordinates, an interbeat interval series, or a hand trajectory. An extracted digital feature is a computed property of that signal, such as frontal theta power, fixation duration, heart-rate variability, path length, or movement smoothness. A digital measure uses one or more features to quantify a clinically relevant construct: for example, cognitive workload or navigation efficiency. It should be described as a candidate digital biomarker only when evidence indicates that it objectively reflects a biological, cognitive, functional, or treatment-related process. The term clinically validated biomarker should be reserved for measures that have undergone adequate analytical and clinical evaluation for their intended context of use.

Validation should begin with analytical reliability: the system must record the same phenomenon consistently across devices, sessions, and operators. Test–retest reliability, calibration, signal-loss thresholds, preprocessing choices, and robustness to motion artifacts should therefore be reported. Reproducibility also requires standardized task parameters because the same feature may have different meanings when immersion, difficulty, controller type, or assistance level changes.

Clinical validity requires associations with established neuropsychological, functional, or biological reference measures. Correlation with a conventional test is not sufficient when the proposed advantage is ecological validity. VR-derived markers should also explain everyday functioning, assistance needs, or ADL. Sensitivity to change must be evaluated against repeated testing and practice effects, and clinically meaningful thresholds should be established rather than relying only on statistically significant group differences. Studies such as those by Kim et al., Tsai et al., and Wang et al. provide promising evidence of discriminative validity, but their derived features require further longitudinal and functional validation before they can be regarded as established clinical biomarkers [8,25,26].

Predictive validity is particularly important for rehabilitation. Candidate biomarkers should forecast treatment response, functional transfer, disengagement, or need for adaptation using data not employed to build the model. Internal cross-validation is insufficient when samples are small or many features are explored; external validation across independent centers, populations, and hardware configurations is needed. Multimodal models should also demonstrate added value beyond simpler behavioral measures. Neuroimaging-based approaches are particularly relevant in this respect because they may identify neural characteristics associated with responsiveness before treatment begins, although their cost and complexity may limit routine deployment [9,44].

Finally, clinical interpretability is essential. A model may classify impairment accurately while remaining unsuitable for rehabilitation if clinicians cannot understand which features drove the prediction or how the result should change treatment. The most useful digital biomarkers will therefore be those that are technically reliable, clinically interpretable, sensitive to meaningful change, and linked to a specific decision, such as increasing difficulty, modifying feedback, identifying fatigue, or predicting transfer to everyday activities.

## 5. Adaptive and Personalized VR Rehabilitation

The next stage in the translational pathway is the use of validated or otherwise interpretable digital measures to modify rehabilitation prospectively. This distinction is important: measuring workload, engagement, fatigue, or neural activity does not by itself constitute closed-loop rehabilitation. A system becomes adaptive only when information obtained during treatment is used to modify subsequent task difficulty, pacing, guidance, feedback, or other intervention parameters. Combining VR with multimodal sensing and artificial intelligence may enable rehabilitation to adapt not only to observed performance but also to the patient’s current state. Integrated behavioral, ocular, motor, autonomic, and neural signals could help identify cognitive overload, fatigue, disengagement, or readiness to progress to a more demanding task.

### 5.1. Multimodal Data Integration

Each sensing modality provides only a partial and potentially ambiguous representation of the patient. Reduced accuracy may indicate insufficient task understanding, memory impairment, distraction, fatigue, or excessive difficulty. Prolonged completion time may reflect cognitive slowing, cautious strategy, motor impairment, or unfamiliarity with the interface. Similarly, increased electrodermal activity or heart rate may represent productive engagement, physical effort, anxiety, discomfort, or cybersickness. Combining modalities can help resolve these ambiguities. For example, declining accuracy accompanied by longer fixations, increased corrective hand movements, and elevated physiological arousal may suggest excessive cognitive demand, whereas declining accuracy associated with reduced exploration and low physiological activation may be more consistent with disengagement.

Multimodal integration may occur at different analytical levels. Individual signals can first be processed into features, such as error rate, gaze entropy, movement smoothness, heart-rate variability, or EEG spectral power, and then combined into a predictive model. Alternatively, separate models can estimate behavioral performance, workload, and engagement before their outputs are integrated into a higher-level decision. Machine-learning methods can identify nonlinear relationships that would be difficult to specify in advance and may allow prediction of treatment response, optimal task difficulty, or imminent performance deterioration. VR–brain–computer interface frameworks have specifically been proposed for the combined training and evaluation of working memory through the integration of task performance and EEG activity [22].

Most current models are trained using group-level data, although the same physiological response may have different meanings across individuals. Age, cognitive reserve, neurological diagnosis, medication, motor impairment, autonomic dysfunction, previous gaming experience, and baseline anxiety can all influence sensor measures. Individualized models should therefore compare current performance with the patient’s own baseline and learning history, rather than relying exclusively on thresholds derived from healthy or diagnostically heterogeneous groups. Such models may require an initial calibration phase followed by continuous updating as the patient becomes familiar with the environment and improves during rehabilitation.

### 5.2. Adaptation and Closed-Loop Systems

A fully closed-loop rehabilitation system can be represented as a continuous cycle. As illustrated in Figure 2, this cycle integrates continuous sensing and patient-state estimation with the subsequent adaptation and reassessment of the intervention.

Adaptation may involve changing the number of stimuli, memory load, presence of distractors, required planning steps, navigation complexity, response window, or degree of dual-task interference. The system may also modify pacing, session duration, the amount of guidance, frequency of prompts, or the form of feedback. A patient showing accurate but effortful performance might continue at the same difficulty with additional time, whereas one showing stable accuracy, efficient exploration, and decreasing physiological effort could progress to a more complex task.

Conversely, signs of fatigue or overload could trigger a reduction in complexity, a brief pause, or a change in feedback rather than simply terminating the exercise.

The CortexVR platform provides a useful methodological example of automated cognitive training based on performance monitoring and reinforcement-learning-supported adaptation, although it was developed for executive-function training in soccer players rather than neurological rehabilitation [27]. In clinical populations, recent evidence supports the feasibility of personalized technology-based cognitive interventions. Puig-Davi et al. compared executive-function-focused immersive VR, adaptive multidomain computerized cognitive training, and standard care in patients with Parkinson’s disease and mild cognitive impairment, demonstrating the increasing clinical maturity of adaptive digital interventions, although the VR arm itself was not a physiologically driven closed-loop system [12].

Physiology-based adaptation remains substantially less developed. Lemay and Levac proposed combining electrodermal activity, heart rate, EEG, eye tracking, behavioral performance, and self-reported states to identify a “just-right” VR challenge level. This approach recognizes that performance-based algorithms alone may fail to capture attention, motivation, workload, and engagement [20,21]. However, converting these associations into reliable real-time adaptation rules requires robust signal quality, individual calibration, temporal synchronization, and validation that the resulting adjustments improve learning rather than merely immediate task performance.

VR-BCI prototypes further illustrate the potential for neural activity to contribute directly to rehabilitation control. Systems have been developed for EEG-supported N-back memory training in dementia and for visually evoked potential-based attention rehabilitation in unilateral spatial neglect, but these remain early technical demonstrations rather than validated clinical interventions [23,24].

Overall, most existing systems are only partially adaptive: difficulty is commonly adjusted between trials or sessions using accuracy and response time, while gaze, autonomic, and EEG signals are mainly recorded for subsequent analysis. The principal challenge is therefore not collecting additional data, but demonstrating that multimodal state estimation can guide transparent, safe, and clinically beneficial adaptations. Human oversight should remain central, particularly when algorithms alter treatment intensity in cognitively impaired or medically vulnerable patients.

## 6. Ecological Validity, Functional Transfer, and Indoor Assisted Living

The final and most clinically demanding stage of the translational pathway is functional transfer. Even technically reliable sensing, valid state estimation, and successful within-session adaptation have limited clinical relevance unless they ultimately improve functioning beyond the trained virtual environment. The clinical value of VR depends not only on immersion or technological sophistication, but on whether tasks reproduce the cognitive demands of everyday life and whether training produces benefits outside the virtual environment. Ecological validity therefore has two distinct components: representativeness, referring to how closely a virtual task resembles real-world activity, and generalizability, referring to whether virtual performance predicts or improves actual functioning. A realistic virtual supermarket may appear ecologically valid, but its clinical significance remains uncertain unless performance relates to shopping ability, independence, or support needs.

### 6.1. Ecologically Valid Virtual Tasks

VR can simulate complex activities that are difficult to reproduce consistently in a clinic. Shopping may require remembering a list, planning a route, resisting distractors, comparing products, managing money, and completing the activity efficiently. Cooking simulations can assess sequencing, prospective memory, divided attention, error monitoring, and safety awareness. Medication-management tasks can incorporate identification, timing, dosage, and responses to missed instructions. Navigation scenarios can examine route learning, spatial memory, landmark use, and adaptation when a familiar path is blocked. Household, social, and financial tasks can similarly reproduce cleaning, packing, appointment preparation, conversation, purchasing, and payment decisions.

These environments allow repeated administration with controlled difficulty while recording not only the final outcome but also routes, action sequences, errors, pauses, prompts, and compensatory strategies. The Virtual Reality Functional Capacity Assessment Tool was developed on this premise, recreating routine activities to measure functional capacity more directly than conventional cognitive tests [45]. Reviews of VR applications based on IADL have subsequently shown growing interest in shopping, cooking, navigation, and other functionally meaningful tasks, although task design and outcome selection remain heterogeneous [46].

Our group used non-immersive VR to train supermarket shopping, suitcase preparation, medication use, and other functional living skills in adolescents and young adults with intellectual disability and in people with major neurocognitive disorders. The home-based study in intellectual disability combined repeated virtual practice with blinded in vivo assessments, demonstrating the feasibility of remote functional training and directly examining generalization beyond the computer task [14]. Subsequent studies showed that people with major neurocognitive disorders could relearn selected functional living skills and that response varied according to clinical and disease-related characteristics [15,16]. These findings illustrate an important advantage of ecologically designed VR: the training target can be defined as an everyday activity rather than an abstract cognitive domain.

### 6.2. Transfer to Everyday Functioning

Evidence of benefit should be interpreted according to the level of transfer achieved. Improvement in the trained virtual task is the most immediate outcome, but it may reflect familiarity with the interface or memorization of a fixed sequence. Near transfer refers to improvement in untrained tasks depending on similar cognitive processes, whereas far transfer involves broader gains in domains not directly practised. More clinically important is transfer to ADL or IADL, followed by maintenance after training ends.

These levels are often conflated. Fewer errors in a virtual kitchen do not necessarily mean that the person can prepare a meal safely at home, and successful headset navigation does not establish independent community mobility. Demonstrating real-world transfer requires outcomes distinct from the trained simulation, such as direct observation, performance-based functional tests, caregiver reports, passive monitoring, or objectively documented changes in assistance needs. Follow-up is also necessary because immediate improvement may not persist.

Some encouraging evidence is available. In adults with intellectual disability, immersive VR training produced greater improvement and retention of a real-world waste-management skill than non-immersive training [39]. Conversely, a recent study found improved cognitive–motor performance after immersive VR but limited transfer to specific real-world tasks, confirming that transfer cannot be assumed from gains within the virtual environment [47]. A systematic review and meta-analysis in older adults with mild cognitive impairment reported benefits of VR for cognition- and IADL-related outcomes, but the evidence remained limited by small samples, heterogeneous interventions, and variable functional measures [48]. Realism may facilitate transfer, but it is not equivalent to demonstrated ecological effectiveness.

### 6.3. Home and Assisted-Living Implementation

Home deployment extends VR from episodic clinic-based treatment to longitudinal rehabilitation and monitoring. Sessions can be delivered more frequently, performance can be observed across weeks or months, and difficulty can be adjusted without repeated travel. The Kinect-based telerehabilitation service described by Kizony et al. [29] showed that remote rehabilitation could be used by people with acquired brain injury at home, with favorable usability and improvements in selected motor and cognitive outcomes. Such programs also expose practical requirements less visible in laboratory studies: reliable installation, remote troubleshooting, adherence monitoring, safety procedures, and interfaces accessible to people with cognitive or motor limitations.

Clinician dashboards are central to this model. They should present interpretable summaries of adherence, errors, prompts, learning trajectories, fatigue, and adverse events rather than streams of raw data. Remote systems developed by Quintana et al. allow clinicians to configure and monitor VR rehabilitation exercises from another location, illustrating how expert supervision can be retained while treatment occurs at home [30,31]. Caregivers may support equipment use, safety, and motivation, but systems should not transfer an excessive technical or supervisory burden to families.

The next step is integration with the wider indoor assisted-living environment. VR-derived measures could be combined with wearable activity, sleep, cardiovascular, or mobility data and with ambient information from smart-home sensors. Mixed-reality systems can also provide graded assistance during actual household tasks; a recent platform guided older adults with and without neurocognitive disorders through activities in a smart apartment while maintaining a low reported cognitive load [40]. Such integration could test whether virtual gains correspond to changes in everyday routines and could trigger clinical review when performance deteriorates. Continuous monitoring nevertheless raises issues of consent, privacy, data ownership, false alerts, and caregiver surveillance. The objective should not be maximal data collection, but a proportionate system that supports independence, identifies meaningful change, and preserves human oversight.

At present, evidence for indoor assisted-living integration can therefore be separated into three progressively more demanding levels. The first, and currently best-established, is home delivery of rehabilitation with remote monitoring and clinician supervision. The second involves context-aware assistance during everyday activities, including graded cueing or mixed-reality support within home-like environments. The third would require integration of rehabilitation-derived digital measures with longitudinal wearable and ambient sensing to detect meaningful changes in everyday function and to adapt support accordingly. Evidence for this fully integrated model remains preliminary: existing studies demonstrate technical feasibility, remote deployment, or assistance during selected functional tasks, but do not yet establish that multimodal biomarker-informed adaptation improves long-term independence, reduces care needs, or prevents functional decline. Indoor assisted living should therefore be regarded as a promising translational endpoint rather than an already validated extension of VR-based cognitive rehabilitation.

## 7. Current Limitations and Future Directions

Despite rapid technological development, the evidence supporting sensor-integrated VR cognitive rehabilitation remains less mature than the sophistication of many proposed systems might suggest. Studies frequently include small and diagnostically heterogeneous samples and are commonly designed as feasibility studies, single-arm interventions, technical demonstrations, or short pilot trials. Although randomized evidence is increasing, differences in clinical populations, intervention intensity, control conditions, VR configurations, and outcome selection continue to limit comparison and quantitative synthesis. Recent reviews in mild cognitive impairment, dementia, stroke, and other neurological populations have repeatedly identified small samples, short treatment periods, limited follow-up, and inconsistent outcome measures as major weaknesses [17,48,49,50,51].

Technological heterogeneity creates an additional source of uncertainty. Studies described as VR rehabilitation may use desktop displays, projection systems, head-mounted devices, commercial games, or custom immersive environments with substantially different levels of interaction and sensory stimulation. Sensor specifications, sampling rates, latency, field of view, controller characteristics, tracking volumes, and assistance algorithms are often incompletely reported. Consequently, an observed effect cannot always be separated from the properties of a particular device or implementation. Future studies should provide sufficiently detailed descriptions of both the rehabilitation intervention and its technological components to permit replication. Existing rehabilitation reporting recommendations offer a useful starting point, but a dedicated minimum reporting set is needed for sensor-integrated VR, including hardware and software versions, task parameters, calibration procedures, signal-acquisition settings, synchronization methods, preprocessing pipelines, missing-data rules, and algorithm updates [52,53].

Multimodal acquisition also introduces technical vulnerabilities. Eye trackers require calibration that may be difficult to maintain in people with visual, attentional, or postural impairment. Motion capture can be affected by occlusion and tracking loss, while EEG, electrocardiography, and photoplethysmography are susceptible to movement and muscle artifacts. Inaccurate temporal synchronization may obscure the relationship between a stimulus, behavioral response, and physiological change. Missing signals are also unlikely to occur randomly: patients with the greatest motor, cognitive, or behavioral difficulties may produce the poorest-quality data. Reporting only successfully recorded sessions may therefore introduce systematic bias. Studies should define signal-quality thresholds, report reasons for missingness, perform robustness analyses, and evaluate sensor performance under realistic movement conditions rather than only in controlled laboratory recordings [54,55,56].

As discussed in Section 4.2, future studies should adopt harmonized terminology distinguishing raw signals, extracted features, digital measures, candidate biomarkers, and clinically validated biomarkers. Verification, analytical validation, and clinical validation should be explicitly matched to the proposed context of use [57,58]. Validation should extend beyond internal classification accuracy. Test–retest reliability should be examined across sessions and devices, followed by multicenter external validation in populations that differ from those used to develop the model. Algorithms should also be compared with simpler behavioral measures to demonstrate their incremental clinical value.

Sensitivity to change is particularly important in rehabilitation, but statistically detectable change should not be equated with clinically meaningful improvement. Thresholds should reflect changes considered important by patients, caregivers, or clinicians and, whenever possible, correspond to greater independence or reduced assistance needs. Continuous digital measures may detect subtle individual changes, but meaningful-change criteria will probably need to account for disease stage, baseline ability, and the intended context of use [59].

Future trials should prioritize untrained functional tasks, objective ADL and IADL outcomes, and follow-up assessments. Wearable or ambient sensing should be used when it can determine whether improvements in virtual navigation, sequencing, medication management, or other trained activities correspond to meaningful changes in everyday routines. Home sensing should nevertheless remain hypothesis-driven and proportionate, because continuous collection of poorly interpretable data may increase burden and surveillance without improving care.

Usability and safety should be treated as clinical outcomes rather than secondary technical considerations. Cybersickness is often mild, including in older adults, but individual susceptibility may restrict session duration or participation [60]. Cognitive impairment can increase difficulties with onboarding, controller use, understanding instructions, and recovering from errors. Caregiver assistance may improve access but can also transfer a substantial technical and supervisory burden to families. Accessible interfaces, supervised familiarization, individualized progression, and systematic reporting of adverse effects and discontinuation are therefore required [17,61].

Finally, privacy, cybersecurity, and algorithmic transparency become increasingly important when behavioral, physiological, neurophysiological, and domestic data are combined. Future systems should minimize data collection, distinguish research from care-related uses, define data ownership and retention, and provide clinically interpretable explanations for adaptations or alerts. Larger adaptive-intervention trials should determine whether explainable, individualized multimodal models improve rehabilitation outcomes compared with simpler and more transparent performance-based algorithms. Progress will depend on standardized reporting, multicenter validation, objective measurement of real-world function, and close collaboration among patients, caregivers, clinicians, engineers, and data scientists.

## 8. Conclusions

The evidence reviewed indicates that VR can serve simultaneously as a therapeutic platform and as an instrumented environment for measuring how patients perform cognitive and functional tasks. During a single session, VR systems can generate continuous information on accuracy, errors, reaction times, navigation strategies, action sequences, gaze behavior, movement patterns, physiological responses, and neural activity. These data may provide a more detailed representation of how a patient performs a task than conventional pre- and post-treatment scores alone.

The integration of behavioral, oculomotor, kinematic, autonomic, and neurophysiological signals offers the possibility of developing more objective and individualized rehabilitation approaches. Multimodal measures may help distinguish cognitive impairment from motor or interface-related limitations, estimate workload and engagement, monitor learning trajectories, and identify when task difficulty or feedback should be modified. In principle, such information could support adaptive and closed-loop interventions tailored to the patient’s changing abilities and state.

However, most variables currently reported in VR studies should be regarded as digital measures or candidate biomarkers rather than clinically validated digital biomarkers. Evidence regarding reliability, sensitivity to meaningful change, predictive validity, external reproducibility, and association with everyday functioning remains limited. Similarly, most adaptive systems rely primarily on accuracy and response time, while real-time adaptation based on physiological or neurophysiological state estimation remains largely experimental.

Future research should therefore prioritize clinical interpretability over technological complexity. Sensor-derived measures should be linked to explicit rehabilitation decisions, validated across centers and devices, and compared with established cognitive and functional outcomes. Most importantly, studies should determine whether improvements within virtual environments translate into sustained gains in ADL and independence. Integration with remote supervision, wearable technologies, and ambient home sensors may ultimately support scalable indoor assisted-living applications, provided that usability, safety, privacy, and human oversight remain central to system design.

## Figures and Tables

**Figure 1:**
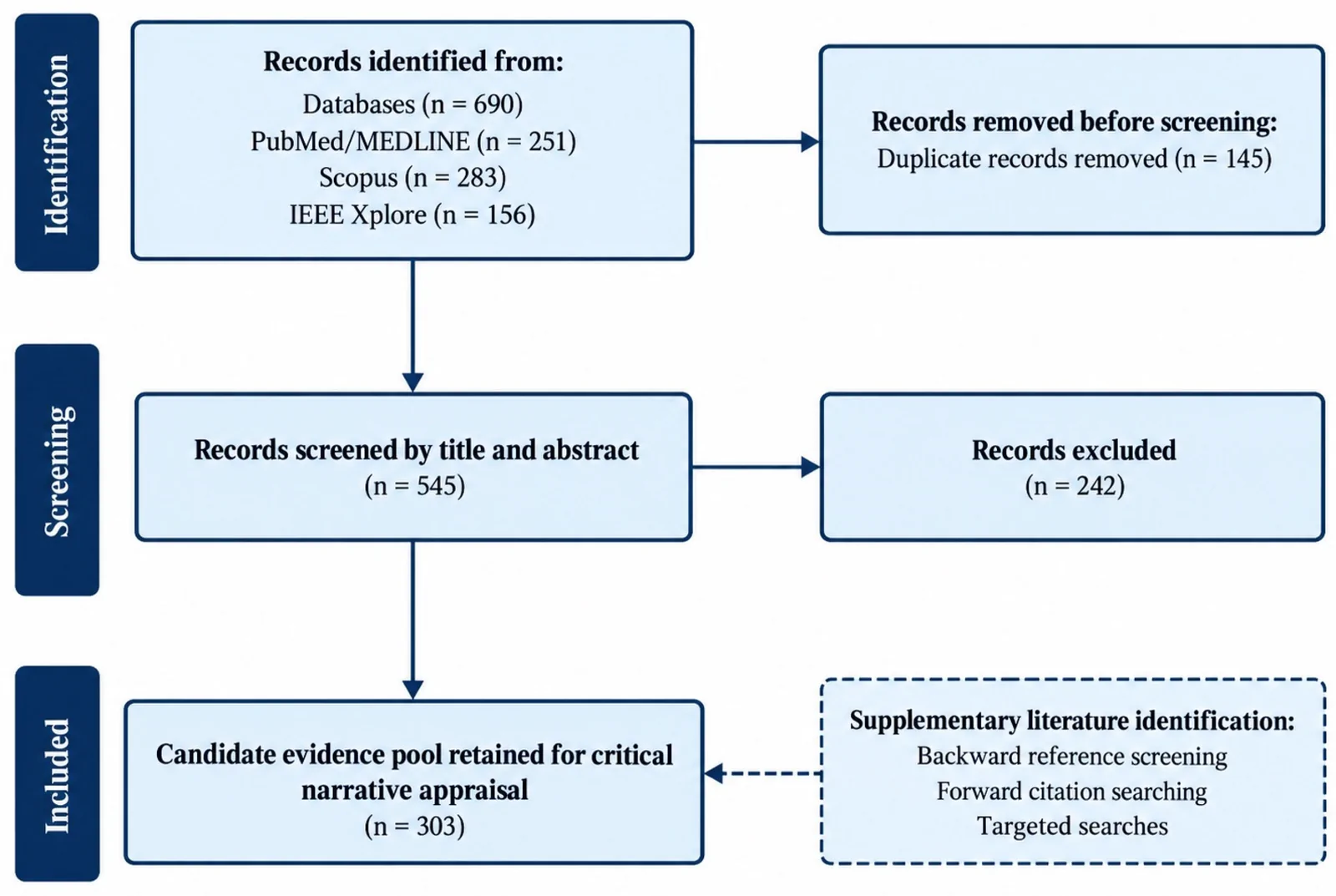
Literature-identification workflow supporting the critical narrative review. Records retrieved from PubMed/MEDLINE, Scopus, and IEEE Xplore were deduplicated and screened by title and abstract for relevance to the predefined review scope.

**Figure 2:**
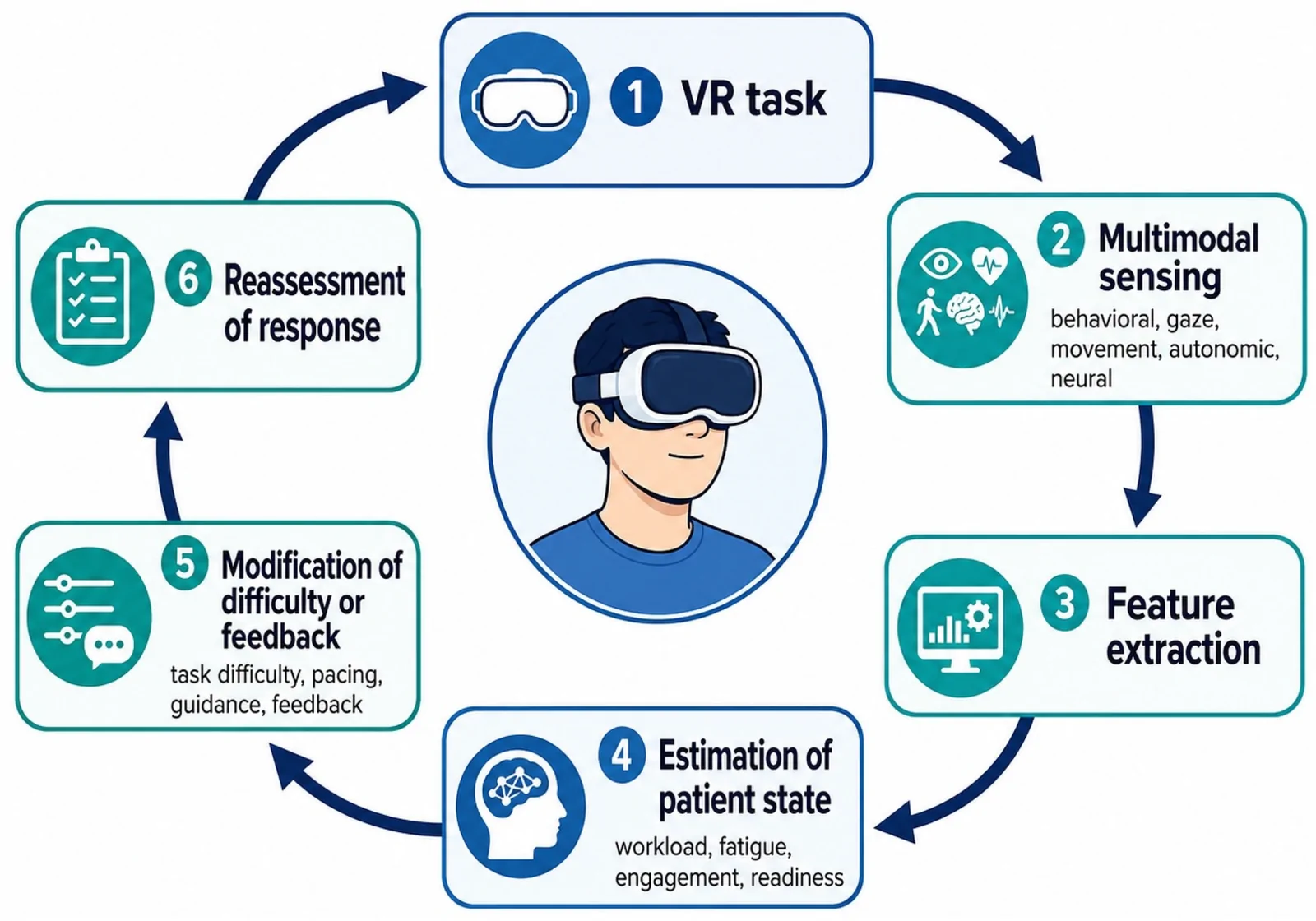
Closed-loop adaptive VR cognitive rehabilitation. The VR task generates multimodal behavioral, ocular, motor, autonomic, and neural data. Extracted features are used to estimate the patient’s current state and modify task difficulty, pacing, guidance, or feedback, after which the response is reassessed and the cycle is repeated.

**Table 1:** Representative evidence across the translational pathway from multimodal sensing to digital-biomarker validation, adaptive rehabilitation, and functional transfer in VR-based cognitive rehabilitation.

Study	Evidence Role/Design and Population	VR and Sensing/Digital Measure	Key Findings	Validation Status	Adaptation Used?	Functional/ADL Transfer?
Cavedoni et al., 2020 [7]	Conceptual perspective focused on MCI	Ecological virtual IADL, behavioral/kinematic measures, clinical data, AI	Proposed integration of VR-derived behavioral and kinematic information with clinical measures to generate multidimensional digital biomarkers	Conceptual framework; no empirical biomarker validation	Proposed only	Not demonstrated
Ansado et al., 2021 [9]	Critical narrative review of neurological cognitive rehabilitation	VR combined with fMRI, EEG and fNIRS	Proposed neuroimaging/neurophysiology as potential predictive markers of response to VR cognitive training	Review-level evidence; no prospective biomarker validation	Proposed only	Not demonstrated
Tan et al., 2021 [10]	Preliminary methodological study in MCI	Scene-based VR memory training with simultaneous EEG	EEG changes across ecologically oriented VR scenes suggested feasibility of objective neurophysiological monitoring during training	Methodological/measurement feasibility	No	Not assessed
Qu et al., 2022 [18]	Experimental multimodal study; 40 younger and older participants	Immersive VR + EEG connectivity + Kinect-recorded movement + behavioral/subjective indices	Age-related alpha/beta connectivity differences and lower speed/acceleration in older participants; several movement variables correlated with cognitive level	Measurement association/feasibility; no clinical biomarker validation	No	Not assessed
Gangemi et al., 2023 [11]	Quasi-randomized trial; 30 patients with chronic ischemic stroke	VR cognitive rehabilitation + EEG theta/alpha/beta analysis	VR was associated with increased occipital alpha and frontal beta activity relative to conventional rehabilitation; behavioral post-treatment outcomes were not assessed	Clinical neurophysiological-response evidence, not biomarker validation	No	Not demonstrated
Maggio et al., 2020 [19]	Single case report; 17-year-old patient in minimally conscious state after severe TBI	Semi-immersive VR + 19-channel EEG; ERS/ERD and ERSP	Concurrent improvements in clinical scales and EEG sensorimotor responses after training	Proof-of-concept case evidence; cannot establish efficacy	No	Not assessed
Kim et al., 2023 [8]	Internal validation; 31 MCI + 20 healthy controls	Virtual kiosk + hand tracking + eye tracking + SVM	Classification reached 93.3% accuracy, 100% sensitivity, 83.3% specificity, 90% precision and 94.7% F1-score	Internal discriminative validation; reliability/external validation required	No	Not assessed
Houzangbe et al., 2024 [20]	Prospective cross-sectional protocol in typically developing children	Progressive immersive-VR difficulty + EDA, HR, EEG, eye tracking, performance and self-report	Protocol designed to determine whether physiology can identify an individually appropriate “just-right” challenge	Protocol only; no outcome evidence	Proposed physiology-driven adaptation only	Not assessed
Houzangbe et al., 2026 [21]	Repeated-measures pilot; 13 typically developing children	Immersive VR + EDA + HR + dry EEG	>90% usable EDA/HR data but only 48.98% usable EEG; normalized EDA increased with task difficulty; performance/self-report differences were nonsignificant	Physiological feasibility evidence; no clinical validation	No; physiological state was measured but did not drive adaptation	Not assessed
Duan et al., 2023 [22]	Perspective/opinion article on BCI-VR working-memory training	VR + EEG; proposed NIRS/fMRI/eye tracking/ECG fusion	Identified real-time neural monitoring, multimodal fusion and closed-loop adaptation as future directions	Conceptual/review evidence	Proposed only	Not demonstrated
Delaux et al., 2024 [23]	Methodology/protocol for chronic unilateral spatial neglect	Standalone VR serious game + dry-electrode VEP-BCI	Proposed portable system combining bottom-up and goal-directed attention training with real-time monitoring of visual attention	Technical/protocol evidence	Proposed BCI-supported adaptive training	Home/self-supervised use proposed, not demonstrated
Angthong et al., 2025 [24]	Preliminary prototype feasibility; 5 healthy young adults	VR N-back + 4-channel OpenBCI EEG; alpha/theta, beta/theta and beta/alpha ratios	Prototype successfully integrated working-memory training and real-time EEG display; descriptive frontal-theta changes accompanied memory-task demands	Prototype feasibility only	Adaptive N-back; EEG feedback was displayed but not validated as adaptation driver	Not assessed
Tsai et al., 2021 [25]	Preliminary diagnostic study; MCI/early AD and small healthy comparator	Immersive virtual supermarket + 46 behavioral indices + ML	45/46 indices differed between groups; classifiers reached up to 100% accuracy	Preliminary internal validation; substantial overfitting risk because of very small comparator sample	No	Not assessed
Wang et al., 2024 [26]	Large internal validation; 301 MCI + 1027 healthy older adults	Immersive virtual supermarket; behavioral, trajectory, regional and cognitive-task data + ensemble ML	Ensemble method outperformed conventional imbalance approaches across recall, F1, AUC and G-mean	Large-sample internal classification validation; external validation still needed	No—adaptive ensemble learning is classifier adaptation, not rehabilitation adaptation	Not assessed
Krupitzer et al., 2022 [27]	Technical/usability study in healthy soccer players	Immersive executive-function training + behavioral monitoring + coach interface + reinforcement learning	Favorable usability; reinforcement-learning component demonstrated feasibility of automated individualized task adaptation	Technical feasibility, not clinical rehabilitation validation	Yes—performance/RL-driven	No clinical/ADL transfer
Puig-Davi et al., 2026 [12]	24-week randomized clinical trial; 51 patients with PD-MCI	Immersive VR vs. adaptive computerized training vs. standard care; cognitive, functional, digital-performance and connectivity outcomes	Both active interventions improved cognitive/functional outcomes vs. standard care; IVR additionally improved dependency/apathy measures; digital and connectivity measures changed	Clinical intervention evidence; digital/neural changes are not validated biomarkers	Adaptive training occurred in the computerized arm; IVR was not physiology-driven closed loop	Functional outcomes improved, but direct real-world ADL transfer was not demonstrated
Cameirão et al., 2016 [28]	Within-subject pilot; 10 stroke survivors	VR cognitive–motor task + camera-based arm tracking + eye tracking	Emotional valence influenced attention/accuracy and false memories; task measures correlated with cognition and depressive symptoms	Clinical multimodal feasibility/association	No	Not demonstrated
Kizony et al., 2017 [29]	Retrospective home telerehabilitation; 82 users, 74 with ABI	Kinect-based 3D tracking + remotely delivered home rehabilitation	High satisfaction/usability; follow-up subgroup showed improvements in upper-limb measures and Trail Making Test (*p* < 0.05)	Clinical/home-deployment feasibility	Program/therapist driven, not sensor-state closed loop	Home delivery demonstrated; direct ADL transfer not specifically tested
Quintana et al., 2022 [30]	Technical evaluation + usability pilot with rehabilitation professionals	Immersive VR authoring system with procedural generation and real-time feedback	Enabled nontechnical clinicians to configure VR rehabilitation scenarios; preliminary usability feedback was favorable	Technical/usability feasibility	Clinician-configurable rather than automated closed loop	Not assessed
Quintana et al., 2024 [31]	Technical system evaluation; remote-monitoring scenarios + developer assessment	Meta Quest 2 + hand tracking + client-server remote monitoring/control	Remote architecture substantially reduced session overhead while maintaining high frame rate and low bandwidth requirements	Technical implementation evidence	Remote clinician adjustment, not physiological closed loop	Remote delivery supported technically; clinical transfer not demonstrated
Wagnerberger et al., 2021 [13]	IK benchmarking + within-subject user study; 10 participants	HMD + hand/full-body tracking + inverse-kinematics avatar reconstruction	VRIK showed mean elbow-positioning error of approximately 5.3 cm; full-body representation increased embodiment and was preferred to hands-only display	Technical/interaction validation	No	Not assessed
Zayed and Reda, 2024 [32]	Secondary multimodal classification study; 16 young adults	HTC Vive Pro Eye; EEG + eye tracking + ECG; CNN	Multimodal model classified six cognitive-training states with approximately 97% validation accuracy and 96.7% average precision/F1-type performance	Internal algorithmic validation; no clinical validation	Potential real-time monitoring only; no demonstrated therapeutic adaptation	Not assessed
Panerai et al., 2023 [16]	Nonrandomized intervention in major neurocognitive disorders	Non-immersive VR training of supermarket use, medication management, information retrieval and suitcase preparation + in vivo testing	Experimental groups improved during VR training and subsequently improved corresponding in vivo functional tasks	Clinical functional-transfer evidence	No automated adaptation	Yes—transfer to real-world/in vivo functional tasks demonstrated
Wen et al., 2020 [33]	EEG algorithm/measurement study during VR spatial-memory training	Multichannel EEG; MPCMI multispectral features + CNN	Early vs. late training stages classified with up to 95% accuracy, particularly using theta–beta2–gamma information	Internal algorithmic validation; proposed EEG marker, not clinically validated biomarker	No	Not assessed
Röhrling et al., 2026 [34]	Technical case study in a VR ADHD serious-game environment	Low-cost 8-channel EEG + Meta Quest 2 + EEGNet	Subject-specific online classification reached up to 90% accuracy; impedance, headset pressure and motion artifacts substantially affected signal quality	Technical feasibility only	Real-time classification, but no validated therapeutic closed loop	Not assessed
Damgaard et al., 2026 [35]	Predictive-biomarker study; 36 cognitively impaired participants with mood or schizophrenia-spectrum disorders	Pretreatment functional/structural MRI + VR cognitive remediation	Lower baseline left superior-frontal activity predicted greater immediate VR-CRT cognitive benefit (β = −1.40, 95% CI −2.45 to −0.35; *p* = 0.01), but not benefit at 3 months	Candidate predictive neural biomarker requiring independent replication	No	Cognitive treatment response assessed; no ADL-transfer demonstration
Maddalon et al., 2026 [36]	Randomized intervention; 46 autistic children	VR social-cognition training with rule-based, human-triggered, random, or no adaptive pauses; behavioral/kinematic monitoring	Social cognition improved over time, but no significant differences among pause strategies	Clinical feasibility of adaptive delivery; no superiority demonstrated	Yes—pacing adaptation, but not physiology-driven	Generalization beyond experimental setting not demonstrated
Deng et al., 2026 [37]	Empirical multimodal study in Alzheimer’s disease	Immersive VR + synchronized EEG and eye tracking + real-time cognitive-load modelling	Closed-loop system dynamically modified task parameters according to estimated cognitive state and maintained participants within the targeted load range	Single-study empirical closed-loop validation; independent clinical replication required	Yes—real-time multimodal state-informed closed loop	Not demonstrated
Caiazza et al., 2026 [38]	Single-blind randomized waiting-list trial; 40 stable outpatients with schizophrenia	12-session immersive VR cognitive rehabilitation	Between-group benefits for negative symptoms (*p* < 0.01), SRT/CRT (*p* = 0.01), DSST (*p* = 0.02) and verbal fluency (*p* = 0.04); GAF and TMT-A nonsignificant	Clinical efficacy/feasibility evidence, not biomarker validation	No sensor-driven adaptation demonstrated	No direct ADL transfer
Franze et al., 2024 [39]	Comparative intervention; 36 adults with intellectual disability	Immersive vs. non-immersive virtual life-skills training	IVR produced better real-world performance after training (d = 1.00) and at 1-week follow-up (d = 1.12)	Functional-transfer evidence	No	Yes—real-world skill transfer and short-term retention demonstrated
Spalla et al., 2026 [40]	Assistive-technology evaluation; 12 older adults with/without neurocognitive disorders	HoloLens 2 mixed-reality assistance with graded cueing during home-relevant activity	System autonomously provided graded assistance with low reported cognitive load and successfully guided almost all participants	Real-world assistive feasibility, not therapeutic biomarker validation	Yes—behavior-contingent graded assistance, not biomarker-driven	Home/ADL-relevant support demonstrated; long-term independence benefit not established

Note: Validation status refers specifically to the evidentiary status of the sensor-derived measure or candidate biomarker and should not be interpreted as a general ranking of study quality. “Adaptation” indicates whether information generated during or immediately preceding the intervention was used prospectively to modify intervention delivery; post hoc machine-learning classification alone was not considered closed-loop adaptation. Functional transfer was considered demonstrated only when outcomes were evaluated outside the trained virtual task.

## Data Availability

No new data were created or analyzed in this study. Data sharing is not applicable to this article.

## Abbreviations

The following abbreviations are used in this manuscript:

ADL	Activities of daily living
AI	Artificial intelligence
BCI	Brain–computer interface
CNN	Convolutional neural network
CRS-R	Coma Recovery Scale–Revised
DoC	Disorders of consciousness
DRS	Disability Rating Scale
ECG	Electrocardiography
EDA	Electrodermal activity
EEG	Electroencephalography
ERD	Event-related desynchronization
ERS	Event-related synchronization
ERSP	Event-related spectral perturbation
ET	Eye tracking
fMRI	Functional magnetic resonance imaging
fNIRS	Functional near-infrared spectroscopy
FPS	Frames per second
GDS-30	30-item Geriatric Depression Scale
HMD	Head-mounted display
HR	Heart rate
IADL	Instrumental activities of daily living
IK	Inverse kinematics
LCF	Levels of Cognitive Functioning
LSL	Lab Streaming Layer
MCI	Mild cognitive impairment
MCS	Minimally conscious state
MMSE	Mini-Mental State Examination
MoCA	Montreal Cognitive Assessment
NIRS	Near-infrared spectroscopy
PC	Personal computer
SSVEP	Steady-state visual evoked potential
TCP	Transmission Control Protocol
TLS	Transport Layer Security
TP-VR	Virtual-reality version of the Toulouse–Piéron cancellation task
USN	Unilateral spatial neglect
VEP	Visual evoked potential
VR	Virtual reality
WMT	Working memory training
XR	Extended reality
c-VEP	Code-modulated visual evoked potential
